# Therapeutic effect of the potent IL-12/IL-23 inhibitor STA-5326 on experimental autoimmune uveoretinitis

**DOI:** 10.1186/ar2530

**Published:** 2008-10-13

**Authors:** Hiroshi Keino, Takayo Watanabe, Yasuhiko Sato, Mamoru Niikura, Yumiko Wada, Annabelle A Okada

**Affiliations:** 1 Department of Ophthalmology, Kyorin University School of Medicine, 6-20-2 Shinkawa, Mitaka, Tokyo, 181-8611 Japan; 2 Division of Radioisotope Research, Kyorin University School of Medicine, 6-20-2 Shinkawa, Mitaka, Tokyo, 181-8611 Japan; 3 Laboratory of Animals, Kyorin University School of Medicine, 6-20-2 Shinkawa, Mitaka, Tokyo 181-8611 Japan; 4 Synta Pharmaceuticals Corporation, 45 Hartwell Ave. Lexington, MA 02421, USA

## Abstract

**Introduction:**

The purpose of this study was to determine if oral administration of the interleukin (IL) 12/IL-23 inhibitor, STA-5326, is effective in experimental autoimmune uveoretinitis (EAU).

**Methods:**

C57BL/6J mice were immunised with human interphotoreceptor retinoid binding protein peptide (IRBP_1–20_). STA-5326 at a dose of either 5 mg/kg or 20 mg/kg, or vehicle alone, was orally administered once a day for six days a week from day 0 to day 14. Fundus examination was performed on day 14 and day 18 after immunisation. Mice were euthanased on day 18 and the eyes were enucleated for histopathological examination. *In vivo*-primed draining lymph node cells were stimulated with IRBP_1–20 _and culture supernatant was harvested for assay of interferon (IFN)-γ and IL-17 by ELISA. Intracellular expression of IFN-γ and IL-17 in CD4^+ ^T cells of cultured draining lymph node cells was assessed by flow cytometry. The level of IL-12 p40 in serum was examined in STA-5326-treated or vehicle-treated mice receiving immunisation.

**Results:**

The level of IL-12 p40 in serum was decreased in mice treated with STA-5326. Oral administration of either 5 mg/kg or 20 mg/kg STA-5326 reduced the severity of EAU on day 14 and 18. In addition, mice treated with 20 mg/kg STA-5326 showed significantly decreased severity of EAU by histopathological analysis. Although IFN-γ production of draining lymph node cells was increased in STA-5326-treated mice by ELISA analysis, the proportion of IFN-γ-producing cells was not significantly altered. However, IL-17 production and the proportion of IL-17-producing cells were significantly reduced in STA-5326-treated mice. Furthermore, oral administration of STA-5326 during the effector phase reduced the severity of EAU.

**Conclusions:**

These results indicate that oral administration of the IL-12/IL-23 inhibitor STA-5326 is effective in suppressing inflammation in the EAU model, and reduces the expansion of IL-17-producing cells. STA-5326 may represent a new therapeutic modality for human refractory uveitis.

## Introduction

Interleukin (IL) 23 is a heterodimeric cytokine, sharing a p40 subunit with the Th1 cytokine IL-12, but differing from IL-12 in its unique p19 subunit [1,2]. IL-23 is required for the generation of effector memory T cells and IL-17-producing T cells (Th17), which in turn play critical roles in inflammatory responses [3,4]. Thus, IL-12/IL-23 has become an attractive clinical target in a number of studies. Investigation into regulation of the p40 and IL-23 specific p19 subunits has demonstrated a critical role of IL-12/IL-23 in the pathogenesis of autoimmune disease [5-9]. Recent studies have demonstrated that monoclonal antibodies to the IL-12/IL-23 p40 subunit are effective in human clinical trials for Crohn's disease and psoriasis [10-12].

Experimental autoimmune uveoretinitis (EAU) is an animal model that shares many clinical and histological features with human uveitic disorders such as Behcet's disease [13-15]. Therefore, much information is gained by using the model to analyse the immunopharmacology of various immunosuppressive agents in uveitis. EAU is induced by immunization with a retinal antigen (S-antigen or interphotoreceptor-retinoid binding protein (IRBP)) or by adoptive transfer of retinal antigen-specific CD4^+ ^T cells [16-18]. Recent studies have demonstrated that a Th1/Th17 response to the retinal antigen is dominant in EAU in mice [19-24]. Although previous reports have stated that IL-12 is required for the induction of EAU [25,26], new research has clearly indicated that it is IL-23, rather than IL-12, that is necessary for EAU induction [24].

The nuclear factor (NF) κB is a popular target for effective blockade of activation of the promoter for genes encoding proinflammatory cytokines in cells involved in innate and adaptive immunity. The NF-κB family includes the p65, RelB, c-Rel, p50 and p52 proteins. Although p50/p65 is the most common form of NF-κB to activate the promoters of many genes, including those for tumour necrosis factor (TNF)-α and IL-6, the c-Rel-containing form is essential for activation of the p40 gene in macrophages [27]. Furthermore, a recent study of the p19 gene promoter showed that c-Rel binds to the κB sites on this promoter and controls p19 gene expression in dendritic cells [28]. Thus, c-Rel is a specific transcriptional regulator of both IL-12 and IL-23.

STA-5326 is a small molecule developed from a novel triazine derivative identified by high-throughout IL-12 inhibitor screening [29]. STA-5326 inhibits the expression of genes encoding the p40 subunit present in both IL-12 and IL-23 by selective inhibition of c-Rel translocation [29]. The protein c-Rel, a member of the Rel/NF-κB family of transcription factors, requires transport from the cytoplasm to the nucleus for activity. STA-5326 blocks the nuclear localization of c-Rel without inhibiting the nuclear import of other Rel/NF-κB family members. Oral administration of STA-5326 led to suppression of inflammation by histopathological analysis in a model of inflammatory bowel disease (IBD) [29]. In the current study, we examined if oral administration of the potent IL-12/IL-23 inhibitor, STA-5326, would be effective in EAU.

## Materials and methods

### Animals

Six- to eight-week-old female C57BL/6J mice were purchased from Japan CLEA (Shizuoka, Japan). All mice were treated in accordance with the ARVO Statement for the Use of Animals in Ophthalmic and Vision Research and institutional guidelines regarding animal experimentation.

### Induction and scoring of EAU

Mice were immunized subcutaneously in the neck region with 200 μg of IRBP_1–20 _emulsified in 0.2 ml of complete Freund's adjuvant (CFA) (Difco, Detroit, MI) containing 1 mg of the *Mycobacterium tuberculosis *strain H37Ra (Difco, Detroit, MI). They were also given 100 ng of pertussis toxin (Sigma, St. Louis, MO) intraperitoneally as additional adjuvant [30]. Funduscopic examination was performed on days 14, 15 and 18 after immunization, and clinical findings were graded from 0 to 4 as previously described [31]. Eyes were enucleated on day 18 and inflammation was assessed histopathologically by scoring on a scale of 0 to 4 in half-point increments, according to a semi-quantitative system [15].

### Oral administration of STA-5326

In most experiments, 5 mg/kg or 20 mg/kg STA-5326 (Synta Pharmaceuticals Corporation, Lexington, MA) or vehicle only (0.5% carboxyl methyl cellulose) was orally administered once a day for six days a week from day 0 to day 14 after immunization. In the effector phase experiments, 20 mg/kg STA-5326 or vehicle was orally administered once a day, from day 9 to day 14 after immunization.

### *In vitro *proliferation and cytokine assay

Cervical lymph node cells obtained from immunized mice on day 18 (2 × 10^5^cells/well) were cultured in 0.2 ml RPMI 1640 (Sigma Aldrich, St. Louis, MO) containing 10 mM HEPES (Invitrogen Life Technologies, Carlsbad, CA), 0.1 mM nonessential amino acid (Invitrogen Life Technologies, Carlsbad, CA), 1 mM sodium pyruvate (Invitrogen Life Technologies, Carlsbad, CA), 100 U/ml penicillin (Invitrogen Life Technologies, Carlsbad, CA), 100 μg/ml streptomycin (Invitrogen Life Technologies, Carlsbad, CA), 1 × 10^-5 ^M 2-mercaptoethanol (2-ME; Sigma Aldrich, St. Louis, MO), 10% FCS, and 10 μg/ml IRBP_1–20_. For cytokine assay, supernatants were collected after 72 hours and analysed for IFN-γ, IL-4 and IL-17 by quantitative capture ELISA using quantikine ELISA kits (R&D Systems, Minneapolis, MN) and mouse IL-17 ELISA Ready-SET-Go kits (eBioscience, San Diego, CA). Cell proliferation was evaluated using a cell proliferation assay (bromodeoxyuridine; Roche Diagnostics, Mannheim, Germany).

### Intracellular cytokine flow cytometry

Cervical lymph node cells obtained from immunized mice were seeded at 1.5 × 10^6 ^cells/well in 24-well plates and stimulated with 10 μg/ml IRBP_1–20 _for 72 hours. The stimulated cervical lymph node cells were harvested and cultured *in vitro *with 5 ng/ml PMA, 500 ng/ml ionomycin and cytokine secretion blocker Gogi-stop (Brefeldin A; BD Bioscience, San Jose, CA) for four hours, then stained using fluorescein isothiocyanate-conjugated monoclonal antibodies against mouse CD4 or CD8 (BD Bioscience, San Jose, CA). The cells were washed, fixed, permeabilised with Cytofix/Cytoperm (BD Bioscience, San Jose, CA), intracellularly stained with phycoerythrin-conjugated antibodies against IFN-γ and IL-17 (BD Bioscience, San Jose, CA) and analyzed on a flow cytometer (FACSCalibur; BD Bioscience, San Jose, CA) using acquisition and analysis software (CellQuest; Becton Dickinson, Franklin Lakes, NJ).

### IL-12 production in the serum of STA-5326-treated or vehicle-treated mice after immunization

Mice were immunized as described above, and 5 mg/kg or 20 mg/kg STA-5326 or vehicle alone was orally administered once a day from day 0 to day 14 after immunization. STA-5326-treated or vehicle-treated mice were euthanased on day 18 after immunization, and serum from individual mice were collected for IL-12 p40 measurement using quantikine ELISA kits (R&D systems, Minneapolis, MN).

### Statistical analysis

Results of experiments were analyzed using Mann-Whitney U test and Student's *t*-test. Data are expressed as the mean ± standard deviation (SD). Means were considered to be significantly different for p < 0.05. All experiments were repeated at least twice, with similar results confirmed.

## Results

### STA-5326 does not alter body weight in EAU mice

**Figure 1:**
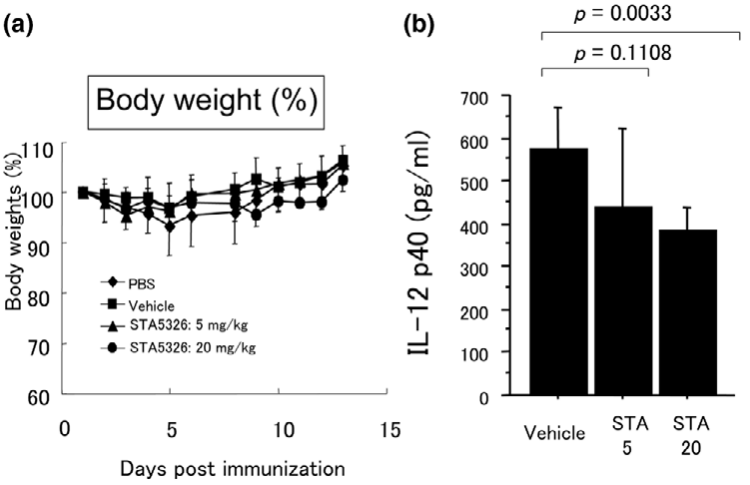
**The level of IL-12 p40 in serum is reduced in STA-5326-treated mice**. (a) STA-5326 at a dose of 5 mg/kg or 20 mg/kg or vehicle only was orally administered once a day six days a week from day 0 to day 14 after immunization with human interphotoreceptor retinoid binding protein (IRBP) peptide, and body weight was measured daily. (b) STA-5326 at a dose of 5 mg/kg or 20 mg/kg or vehicle only was orally administered from day 0 to day 14 after immunization. Individual mice sera were collected on day 18 after immunization and the level of IL-12 p40 was assayed by ELISA. Statistical analysis was performed using Student's *t*-test.

To assess for possible toxicity of STA-5326, the body weight of mice was measured every day after immunization with IRBP peptide and CFA. Body weight did not change in either 5 mg/kg or 20 mg/kg STA-5326-treated or vehicle-treated mice during administration (Figure 1a).

### The level of IL-12 p40 in serum is decreased in STA-5326-treated mice

STA-5326 has been reported to inhibit the expression of genes encoding the p40 subunit present in both IL-12 and IL-23 [29]. To determine if oral administration of STA-5326 changes the level of IL-12p40 *in vivo*, we examined the level of IL-12 p40 in serum. STA-5326 at a dose of 5 mg/kg or 20 mg/kg or vehicle only was orally administered from day 0 to day 14 after immunization. Serum from individual mice was collected on day 18 after immunization, and IL-12 p40 was assayed by ELISA. The level of IL-12 p40 was reduced in STA-5326-treated mice, particularly in the high-dose group, compared with vehicle-treated mice (Figure 1b). These data indicate that oral administration of STA-5326 is capable of reducing the level of IL-12 p40 *in vivo*.

### Oral administration of STA-5326 reduces the severity of EAU by clinical and pathological analysis

**Figure 2:**
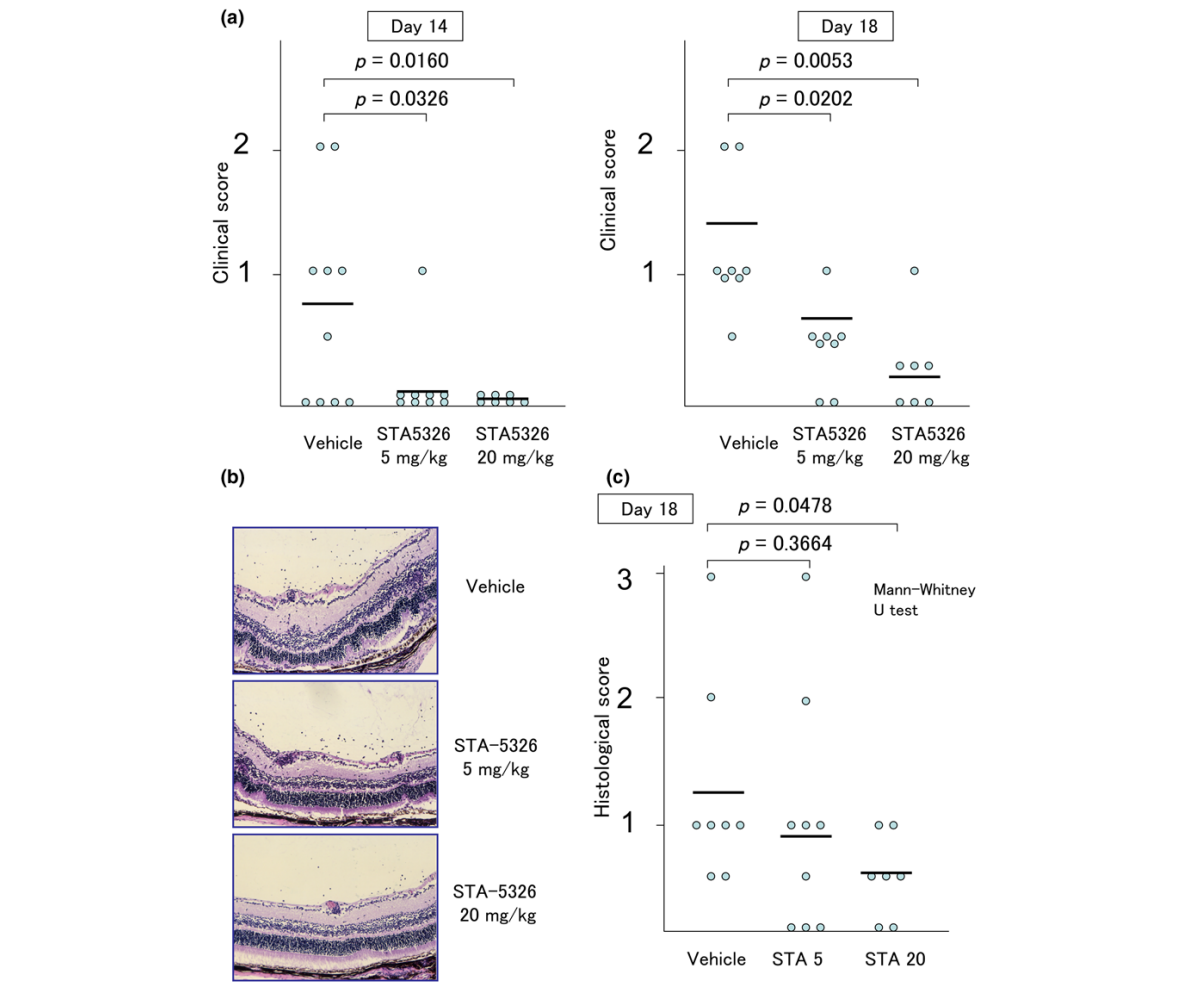
**Oral administration of STA-5326 reduces the severity of experimental autoimmune uveoretinitis (EAU) by clinical and histopathological analysis**. (a) Clinical score of EAU in STA-5326-treated or vehicle-treated mice. Immunized mice were treated with 5 mg/kg or 20 mg/kg STA-5326 or vehicle from day 0 to day 14 after immunization. EAU findings were evaluated on day 14 and day 18 after immunization. (b) Histopathological images of eyes from STA-5326-treated or vehicle-treated mice. Eyes enucleated on day 18 after immunization from STA-5326-treated or vehicle-treated mice were examined. Magnification ×400. (c) Histopathological score of EAU in STA-5326-treated or vehicle-treated mice. Each point on the graph represents the clinical or pathological score of one mouse. Each bar on the graph represents the average clinical or histopathological score for each group. Statistical analysis was performed by Mann-Whitney U test.

We confirmed that STA-5326 treatment decreases the level of Il-12 p40 *in vivo*, and we next tested if oral administration of STA-5326 is effective in EAU. C57BL/6 mice were immunized with 200 μg human IRBP peptide 1–20 and treated with 5 mg/kg or 20 mg/kg STA-5326 or vehicle only from day 0 to day 14 after immunization. The incidence and severity of EAU in STA-5326-treated or vehicle-treated mice were evaluated on days 14 and 18 after immunization. Fundus examination revealed that the severity of EAU was ameliorated in STA-5326-treated mice compared with vehicle-treated mice (Figure 2a). Histopathological examination of eyes from vehicle-treated mice showed severe inflammatory changes. Inflammatory cell infiltration into the vitreous cavity and throughout all layers of the retina, with intensive retinal vasculitis and partial destruction of the retinal architecture, was observed (Figure 2b). In contrast, STA-5326-treated mice exhibited some inflammatory cell infiltration into the vitreous cavity, but only a few infiltrating cells in the retina with retinal layers remaining intact (Figure 2b). This effect on reducing inflammation was dose-dependent, with substantial suppression observed with a dose of 5 mg/kg and stronger suppression observed with a dose of 20 mg/kg (Figure 2c). These results clearly indicate that oral administration of SAT-5326 is effective in suppressing inflammation in EAU.

### IL-17 production and the proportion of IL-17-producing cells of draining lymph nodes are significantly reduced in STA-5326-treated mice

**Figure 3:**
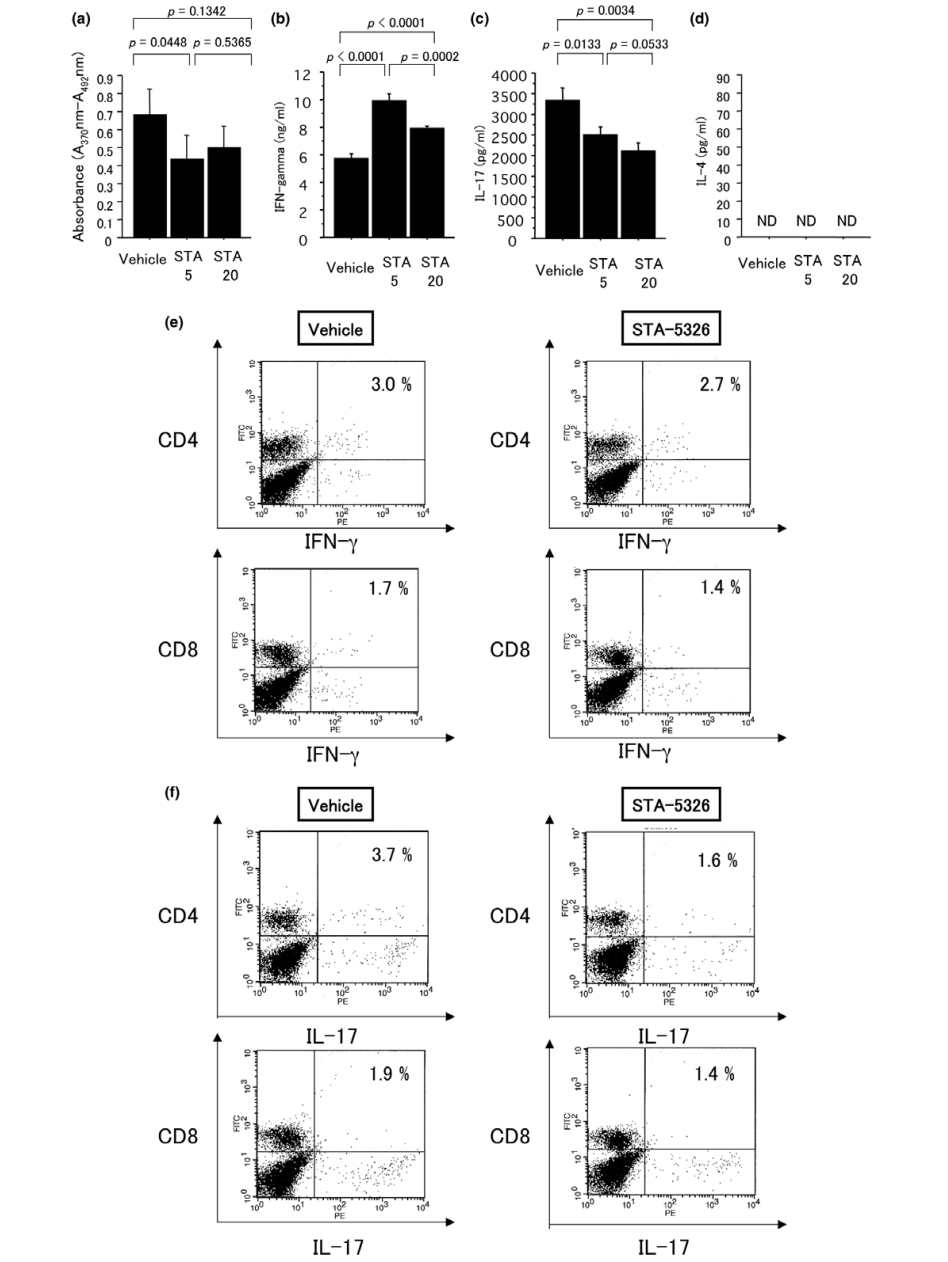
**Antigen specific proliferation is decreased in lymph node cells of STA-5326-treated mice, and IL-17 production and the proportion of Th17 cells from draining lymph nodes are significantly reduced in STA-5326-treated mice**. (a) Antigen specific proliferation of draining lymph nodes in STA-5326-treated or vehicle-treated mice. Immunized mice were treated with 5 mg/kg or 20 mg/kg STA-5326 or vehicle from day 0 to day 14 after immunization. Draining lymph node cells collected on day 18 after immunization were pooled within each group. Cultures were stimulated with 10 μg/ml human experimental autoimmune uveoretinitis (IRBP) peptide 1–20 for 72 hours and pulsed with bromodeoxyuridine for the last 24 hours. (b-d) Cytokine production of interferon (IFN) γ, interleukin (IL) 17 and IL-4 by draining lymph node cells from STA-5326-treated or vehicle-treated mice. Immunized mice were treated with 5 mg/kg or 20 mg/kg STA-5326 or vehicle from day 0 to day 14 after immunization. Draining lymph node cells collected on day 18 after immunization were pooled within each group. Cultures were stimulated with 10 μg/ml IRBP_1–20 _for 72 hours, and supernatants collected at 72 hours were assayed by ELISA. (a-d) Statistical analysis was performed using Student;s *t*-test. (e and f) Intracellular cytokine staining of draining lymph node cells in 20 mg/kg STA-5326 or vehicle-treated mice. Draining lymph node cells collected on day 18 were stimulated with IRBP_1–20 _for 72 hours, and the cultured cells were incubated with PMA plus ionomycin and brefeldin A and stained with CD4, CD8 and intracellular IFN-γ and IL-17. The percentage shown in the upper right quadrant is for IFN-γ or IL-17 positive cells in CD4^+ ^T cells.

STA-5326 is a potent IL-12/IL-23 inhibitor, so we examined antigen-specific proliferation and cytokine production of IFN-γ, IL-17 and IL-4. Immunized mice were treated with 5 mg/kg or 20 mg/kg STA-5326 or vehicle only from day 0 to day 14, and draining lymph node cells were collected on day 18 after immunization and pooled within each group. The cells were stimulated with 10 μg/ml human IRBP peptide 1–20 for 72 hours and pulsed with bromodeoxyuridine for the last 24 hours. The proliferation of antigen-specific cells from STA-5326-treated mice was reduced compared with that from vehicle-treated mice (Figure 3a). Next, draining lymph node cells collected on day 18 after immunization were stimulated with 10 μg/ml human IRBP peptide 1–20 for 72 hours, and supernatants collected at 72 hours were assayed by ELISA. Lymph node cultures from STA-5326-treated mice showed elevated production of IFN-γ and decreased production of IL-17 compared with those from vehicle-treated mice (Figures 3b and 3c). IL-4 was not detected in either group (Figure 3d). The population of IFN-γ-producing or IL-17-producing draining lymph node cells from mice treated with 20 mg/kg STA-5326 or vehicle-treated mice was also examined. Cultured lymph node cells were stimulated with PMA/ionomycin, followed by intracellular staining for IFN-γ and IL-17. Although the number of IFN-γ -producing CD4^+ ^T cells (Th1 cells) was the same in mice treated with STA-5326 or vehicle only, the number of IL-17-producing CD4^+ ^T cells (Th17 cells) was reduced in STA-5326-treated mice (Figures 3e and 3f). The number of IFN-γ-producing and IL-17-producing CD8^+ ^T cells was no different between the two groups (Figures 3e and 3f). These data demonstrate that administration of STA-5326 during the entire phase of EAU reduces the expansion of Th17 cells, but not Th1 cells.

### Oral administration of STA-5326 during the effector phase reduces the severity of EAU by clinical analysis

**Figure 4:**
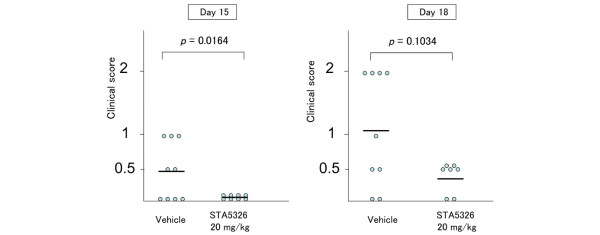
**Oral administration of STA-5326 during the effector phase reduces the severity of experimental autoimmune uveoretinitis (EAU) by clinical analysis**. Clinical score of EAU in STA-5326-treated or vehicle-treated mice. Immunized mice were treated with 20 mg/kg STA-5326 or vehicle from day 9 to day 14 after immunization. Clinical findings were evaluated on day 15 and day 18 after immunization. Statistical analysis was performed by Mann-Whitney U test.

IL-23 is known to be required for the promotion of Th17 cells *in vivo*, [4] so we examined if STA-5326 was effective not only when administered throughout the entire phase of EAU, but also after effector T cells had already been generated. Immunized mice were treated with 20 mg/kg STA-5326 or vehicle only from day 9 to day 14 after immunization (the effector phase). EAU severity was evaluated on day 15 and day 18 after immunization. Fundus examination revealed that STA-5326 treatment significantly reduced the severity of EAU on day 15 (Figure 4). This suggests that oral administration of STA-5326, even during the effector phase, is only capable of decreasing inflammation in EAU.

## Discussion

In the present study, we showed that oral administration of the potent IL-12/IL-23 inhibitor, STA-5326, reduces the severity of EAU by clinical and histopathological analysis. In STA-5326-treated mice, the serum level of IL-12 p40 was decreased. Although antigen specific IFN-γ production was not inhibited in draining lymph node cells from STA-5326-treated mice, IL-17 production and the proportion of IL-17-producing cells were significantly reduced. Furthermore, oral administration of STA-5326 significantly ameliorated the severity of EAU even after effector cells were presumably generated.

Production of the Th1 cytokine, IFN-γ, was not reduced in STA-5326-treated mice. This was not expected, because reduced IFN-γ production had been observed in an animal model of IBD treated with STA-5326 [29]. It is possible that decreased IL-17 production may be affecting IFN-γ production during the late phase of EAU. Luger and colleagues showed that an enhanced Th1 response was observed in lymph node cells of IL-17 knockout mice, suggesting that IL-17 may have an antagonistic effect on the development of Th1 effectors [24]. Yoshimura and colleagues also demonstrated that anti-IFN-γ and anti-IL-4 antibody treatment augmented Th17 differentiation *in vivo *[23]. Therefore, IL-17 and IFN-γ may be acting in a reciprocal fashion during the late phase of EAU, resulting in an absence of down-regulation of IFN-γ due to reduced IL-17 production in STA-5326-treated mice.

Oral administration of STA-5326 during the effector phase only resulted in decreased EAU inflammation on clinical analysis. It has recently been shown that systemic neutralization of IL-23 does not reverse EAU during the effector phase in B10.RIII mice [24]. IL-23 is required for expansion and maintenance of Th17 effectors [4]. In addition, Tarrant and colleagues clearly demonstrated that endogenous IL-12 plays a role in pathogenesis during the expression phase of EAU, after uveitogenic effector cells have been primed [25]. Down-regulation of the IL-12/Th1 pathway and the IL-23/Th17 pathway may be necessary for significant amelioration of EAU during the effector phase and the induction phase, respectively. This would explain why STA-5326, by blocking both IL-12 and IL-23, is efficacious in both phases.

Although the present study examined clinical and histopathological changes only on day 14 and day 18 when STA-5326 was administered during the entire course of EAU, the possibility exists that STA-5326 may be merely delaying the onset of EAU rather than decreasing the overall severity of inflammation. However, STA-5326 also decreased inflammation when administered only during the effector phase of EAU, after the presumed production of uveitogenic effector cells and migration of these cells to the eye. This suggests that STA-5326 may have a therapeutic effect on ocular inflammation in the clinical setting.

Our data are in agreement with previous reports showing that IL-12 p40 knockout and IL-23 p19 knockout mice are highly resistant to EAU and anti-IL-12 antibody treatment prevents EAU induction [24-26]. The present study showed that serum levels of IL-12 p40 were reduced in STA-5326-treated mice in a dose-dependent manner. IL-12 and IFN-γ are known to be important cytokines for host defense and immune surveillance. Recently, the IL-23/IL-17 pathway has been shown to be necessary for host defence against *Klebsiella pneumoniae *[32,33]. p40 is a subunit of both IL-12 and IL-23, and decreased levels of IL-12 p40 may affect a broad range of immune responses. The present study showed that production of IFN-γ from draining lymph node cells was not decreased in mice treated with STA-5326 through the entire course of EAU. Therefore, the safety of STA-5326 administration would need to be assessed further in terms of effect on host defence mechanisms.

It has been demonstrated that STA-5326 inhibits the expression of genes encoding the p40 subunit present in both IL-12 and IL-23 by way of selective inhibition of c-Rel translocation [29]. However, in the present study, it is not clear where SAT-5326 is having this effect. STA-5326 has been reported to inhibit IL-12 production by human monocytes, monocyte-derived dendritic cells, and the human monocyte cell line THP-1 [29]. Serum levels of IL-12/IL-23 p40 were significantly decreased in STA-5326-treated mice in the current study, so STA-5326 may be blocking activation of the promoter for IL-12/IL-23 p40 in macrophages and dendritic cells in lymph nodes, spleen and blood. It is also likely that STA-5326 blocks c-Rel translocation in ocular tissues and infiltrating inflammatory cells, including ocular antigen presenting cells, resulting in inhibition of gene expression of IL-12/IL-23 in the uveitic lesion.

It has been reported that STA-5326 does not affect the production of inflammatory cytokines, including IL-1β, IL-2, IL-4, IL-6, IL-8 and IL-18, in an IFN-γ stimulated human monocytic cell line and in mouse spleen cells [29]. Although the NF-κB family p50/p65 is the most common form of NF-κB to activate the promoters of many genes, including those for TNF-α and IL-6, the c-Rel-containing form is essential for activation of the p40 gene in macrophages [27]. Furthermore, it has recently been shown that c-Rel binds to the κB sites on this promoter and controls p19 gene expression in dendritic cells [28]. Thus, c-Rel appears to be a specific transcriptional regulator for both IL-12 and IL-23. The present study also showed that serum levels of IL-12 p40 were decreased in STA-5326-treated mice. Taken together, we believe that STA-5326 represents a potent IL-12/IL-23 inhibitor.

Compared with anti-cytokine antibodies that act by neutralization of IL-12 and IL-23 proteins that have already been produced, STA-5326 acts by selectively shutting off transcription of the p35, p40 and p19 genes [29] and has the added advantage of being a small-molecule that can be administered orally. Therefore, we believe that STA-5326 has great potential as a therapeutic agent. Accordingly, a biomarker study in which patients with stable psoriasis vulgaris skin plaques received oral STA-5326, showed that expression of IL-23 p19 and IL-12/IL-23 p40 was reduced [34], and STA-5326 is currently undergoing evaluation in a phase 2a study in rheumatoid arthritis, a disease characterized by elevated IL-12 levels.

## Conclusions

Oral administration of STA-5326 was effective in suppressing inflammation in the EAU model, and reduced the serum level of IL-12/IL-23 p40 and the expansion of IL-17-producing cells. STA-5326 represents a new promising therapeutic modality for refractory uveitis in humans.

## Abbreviations

CFA: complete Freund's adjuvant; EAU: experimental autoimmune uveoretinitis; ELISA: enzyme linked immunosorbent assay; RPMI: Roswell Park Memorial Institute; HEPES: 4-(2-hydroxyethyl)-1-piperazineethanesulfonic acid; PMA: Phorbol 12-Myristate 13 Acetate; FCS: fetal calf serum; IBD: inflammatory bowel disease; IFN: interferon; IL: interleukin; IRBP: interphotoreceptor retinoid binding protein; NF: nuclear factor; SD: standard deviation; Th: T helper; TNF: tumour necrosis factor.

## Competing interests

YW is an employee of Synta Pharmaceuticals Corporation. There were no competing interests for the remaining authors.

## Authors' contributions

HK contributed to the design of the study, performance of *in vivo *experiments, data analysis and manuscript preparation. MN contributed to performance of *in vivo *experiments. YS and TW performed the *in vitro *experiments. YW provided vital reagents and contributed to data analysis and manuscript preparation. AAO contributed to data analysis and manuscript preparation. All authors read and approved the final manuscript.
